# Validity and reliability of arm abduction angle measured on smartphone: a cross-sectional study

**DOI:** 10.1186/s12891-016-0957-3

**Published:** 2016-02-20

**Authors:** Antonio I. Cuesta-Vargas, Cristina Roldán-Jiménez

**Affiliations:** 1 Departamento de Fisioterapia, Facultad de Ciencias de la Salud, Universidad de Málaga, Andalucia Tech, Instituto Investigacion de Biomedicina de Málaga (IBIMA), Grupo de Clinimetria (AE-14), Malaga, Spain; 2 School of Clinical Science, Faculty of Health Science, Queensland University Technology, Brisbane, Australia

**Keywords:** Range of motion, Reliability, Measurement, Smartphone, Inertial sensor

## Abstract

**Background:**

Measuring range of movement is important in clinical shoulder assessment. Over the years, different techniques have been used to analyze upper limbs mobility. Smartphone image-based goniometer offers a noninvasive easy-to-use method of measuring arm abduction angle. However, the validity of this method has not been previously established. The purpose of this study was to investigate the validity and reliability of an Internet and image-based app (mROM) regarding arm abduction angle in both healthy subjects and patients suffering from shoulder damage.

**Methods:**

Twenty three subjects with shoulder pathology (14 female, 9 male) and 14 healthy subjects (8 female, 6 male) were examined (37 shoulders). mROM app was used to measure arm abduction angle. Two examiners measured 37 shoulders on 3 separate occasions over 2 days: 2 measurements on the first day and a third one the following day. Descriptive statistics were calculated for descriptive and anthropometric variables, as well as for the first measure of arm abduction angle by photographs and inertial sensors. Reliability was investigated by intraclass correlation coefficients and *p* values, and validity by Pearson correlation and P.

**Results:**

Intra-rater and inter-rater reliability were high (intraclass correlation coefficients 0.998 and 0.984 respectively) for the total sample, although, for the healthy group, intrareliability was lower and interreliability was no reliable. Measurements from photographs and intertial sensors were highly correlated (Pearson *r* = 0.964) for the total sample. However, it was no significant for the healthy group.

**Conclusion:**

Smartphone photographs are a reliable and valid method to measure arm abduction angle, supporting the use of photography obtained through app for measuring joint ROM. This method provides a convenient and precise tool in assessment of arm motion.

## Background

Arm range of motion (ROM) is a measure of interest in clinical setting as it is important for the diagnosis, evaluating the treatment and quantifying possible changes [1]. One of the most widely used methods for evaluating ROM is manual goniometry [2], which was first used to treat injured soldiers during the First World War [3], and has been developed ever since [4, 5]. For decades active and passive upper limb motion has been studied by goniometry [2, 6–9]. Subsequently, ROM has been studied by digital goniometer [10], digital inclinometer [[11, 12]], visual estimation [13], visual level [14] and other devices such 3D gyroscope [15, 16] or Kinect system [17]. Given their compact size and portability, one attractive option is inertial sensors, which are a valid and reliable motion analysis system [18]. Use of inertial sensors is described in a protocol which analyses upper-limb movements [19], whilst their intra- and inter-operator reliability has been determined in several planes of motion [20].

The emergence of new technologies has led to conventional rehabilitation services incorporating the concept of telerehabilitation as an attractive alternative to providing at-distance rehabilitation over the Internet. This brings a wide range of benefits for both patients and health care practitioners, and also improves the quality of rehabilitation health care [21, 22]. There are several studies that have contributed to accepting telerehabilitation as a feasible tool, like those analysing knee angles and kinematic gait by internet-based evaluation [23, 24], and to accepting goniometry over the Internet as a powerful, valid and reliable tool for measuring ROM in joints [25]. In this field, Smartphones are currently very popular devices for therapeutic purposes [26]. Smartphone applications (apps) have lately transformed the mobile phone into a health care provider’s device [27]. Hence, in the past years, health apps that measure ROM have been validated; several apps make use of goniometry in knee [28] or elbow [29] joints. Regarding the shoulder, sensor-based [30–32] and inclinometer-based [33, 34] apps have investigated its motion. Studies have also investigated internet-based goniometers through images of the elbow joint [29, 35, 36] hallux valgus [37] or knee joint [23]. More specifically, the Internet-based goniometer has also been demonstrated to be a reliable tool for the measurement of upper limb ROM in stroke patients [32]. However, only shoulder external rotation through the image-based app has recently been validated in a healthy sample [34]. Interest in analysing upper extremity biomechanics also includes individuals suffering from shoulder diseases, given that the shoulder is the most affected region in the upper limbs after the hand [38]. In patients with chronic shoulder pain, ROM is limited and results in restricted daily activities [39]. Consequently, it is necessary to find simple, effective tools that can improve diagnosis and outcome assessment [38]. Furthermore, rehabilitation services that are technology-focused contemplate the term ‘image-based telerehabilitation’ [40]. Accordingly, the aim of the present research is to study the validity and reliability of an Internet and image-based app regarding arm abduction angle (AAA) in both healthy subjects and patients suffering from shoulder damage.

## Methods

### Study design

In this cross-sectional study, descriptive and anthropometric independent variables related to age, gender, weight, size and BMI were included, along with a physical property for a dependent variable, namely arm abduction angle, AAA (degrees).

Following recruitment, participants were asked to attend the study in the Human Movement Laboratory, Faculty of Health Sciences (University of Málaga). Tasks were explained concisely and clearly so that the participant understood the action to perform. The beginning and the end of the action were determined by a verbal instruction from the researcher. Participants were placed standing, starting from neutral position, performing arm abduction.

### Participants

The total sample consisted of 37 subjects: 23 in the pathologic group and 14 in the healthy group. Patients were recruited from a specialized orthopaedics clinic where they had been previously been diagnosed by magnetic resonance imaging. Asymptomatic subjects were recruited thought advertisement. They were interested in taking part in the project and they met the inclusion criteria. Subjects were included if they were aged between 18 and 75 years old, had a Body Mass Index (BMI) between 18 and 42. Subjects were excluded if they refused to participate in the study.. Asymptomatic subjects were excluded if they had any shoulder pain or they presented a positive Neer [41] or Hawkins [42] test.

Priori sample size was calculated in 9 patients for an α error of 0.05, a statistical power of 0.8 and β error of 0.7, based on data from a systematic review on the use of inertial sensors to measure human movement [18].

### Ethics

Ethical approval for the study was granted by the Ethics Committee of the Faculty of Health Sciences, University of Malaga. The study complied with the principles laid out in the Declaration of Helsinki. Each participant was given an information sheet and provided written informed consent for participation. Participants were informed that participation was voluntary and they could withdraw at any point. They were also assured that their personal data would be treated in accordance with the Organic Law of Protection of Personal Data.

### Apparatus

**Fig. 1:**
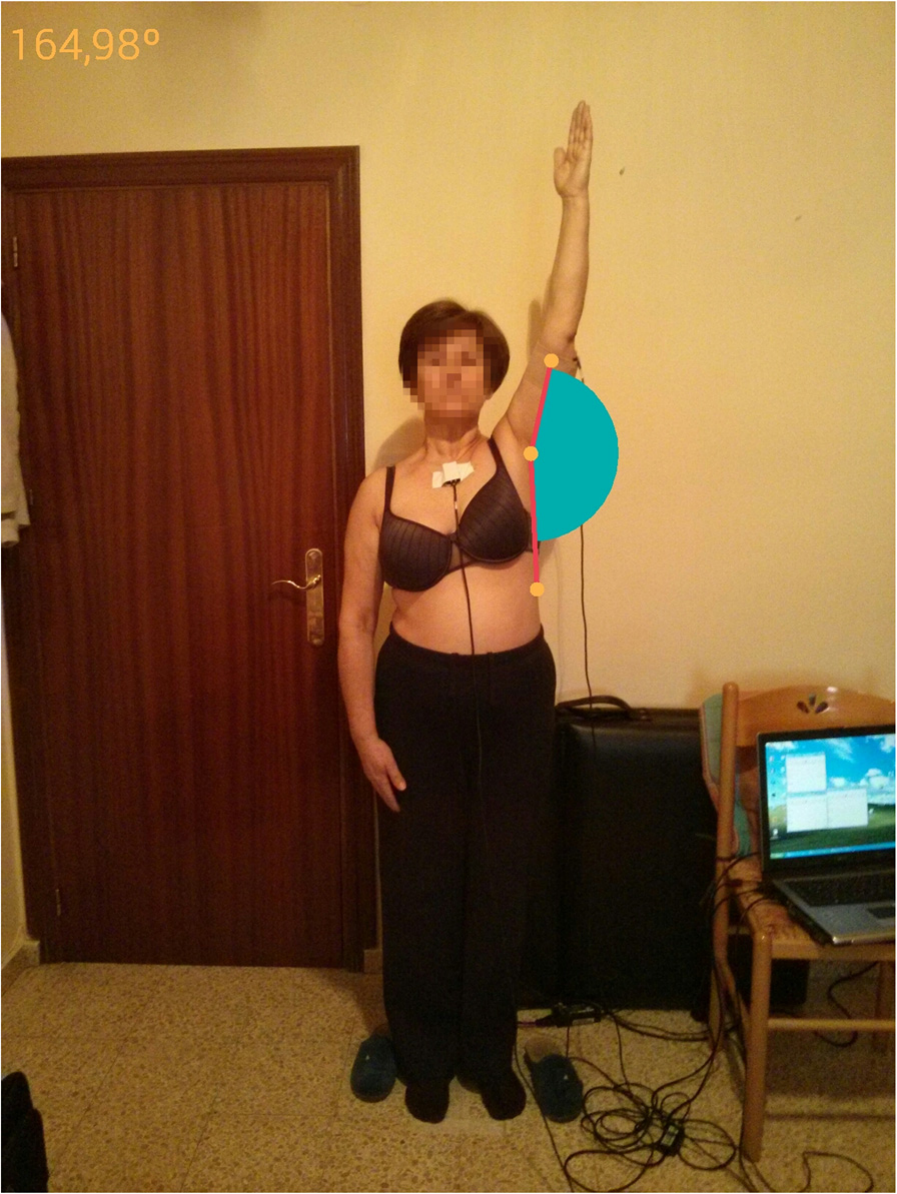
Arm abduction angle measurement made by mROM app. In the arm measured, it can be observed the 3 point as reference to create the angle. In the upper left corner, angle´s degrees are provided automatically by the app. (The subject provided consent for her image to be used)

**Fig. 2:**
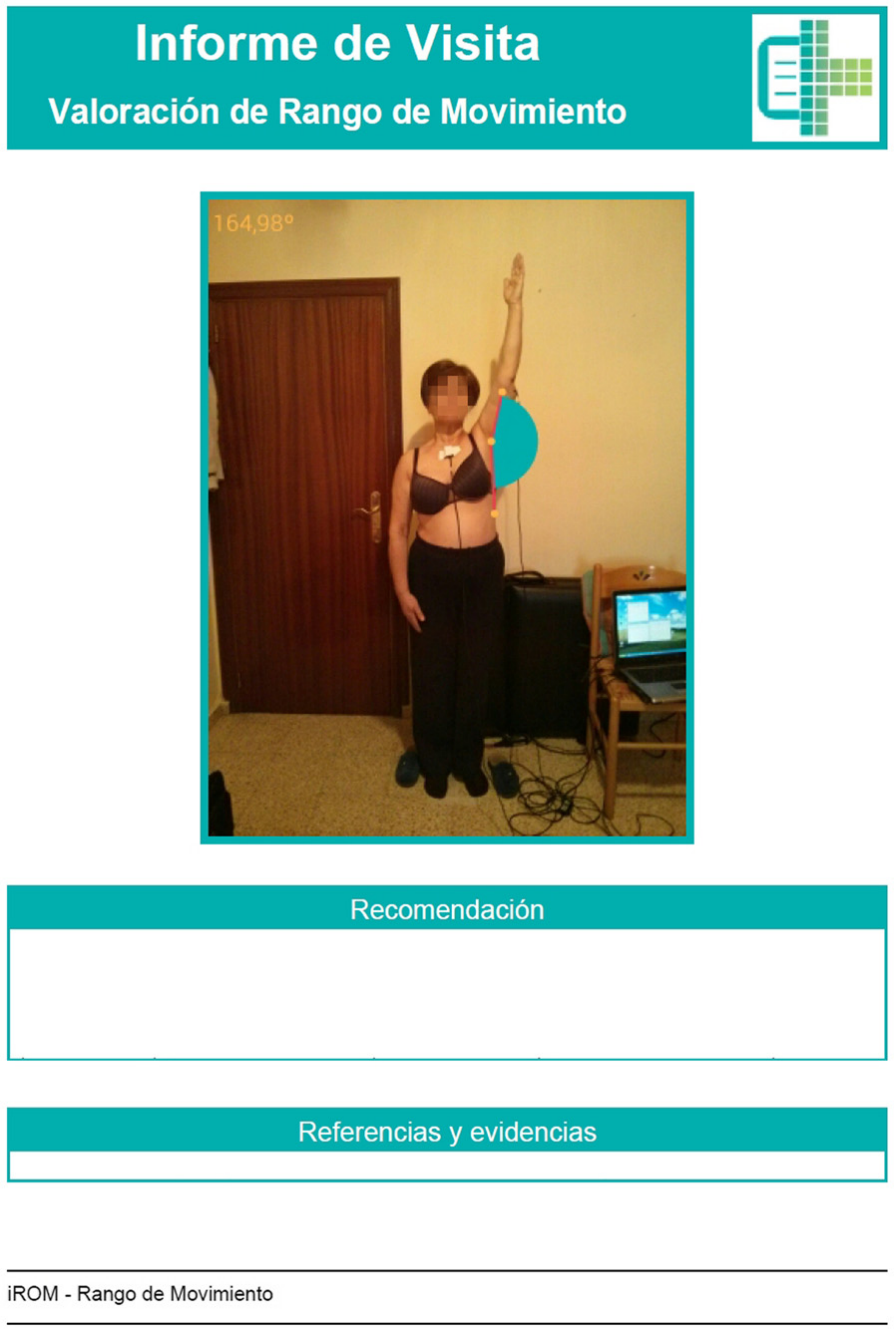
Report with recommendations for clinical use for patients. In the upper part, “Visit report” and “Range of Motion assessment” is heading the report. It is followed by the measurement, a · recommendation” section for clinical use for patients. Finally, there is a “References and Evidences” section, so clinical recommendation can be supported by a medical reference. (The subject provided consent for her image to be used)

AAA was obtained through two different devices. On the one hand, as criterion standard, the inertial measurement was obtained through two inertial sensors (InertiaCube3™ Intersense Inc., Billerica, Massachusetts) with dimensions of 26.2 mm x 39.2 mm x 14.8 mm and weighing 17 grams. Each sensor contains an inertial 3-DOF (Degree of Freedom) orientation tracking system: yaw, pitch, and roll, with an accuracy of 1°, 0.25°, and 25° respectively, an angular range of 360°, able to detect an angular rate between 0° and 1200° per second, with a sampling frequency of 1000 Hz. On the other hand, degrees were also obtained using a Smartphone Nexus 4 ® (LG Electronics INC, Seoul, South Korea) with an 8 megapixels main camera and a 4.7 in. Corning Gorilla Glass 2 touchscreen with a 1280 × 768 pixel resolution. The app used was mROM (Brain Dynamics SL, Málaga, Spain), available in the Google store. mROM is an app that allows ROM to be measured with the camera on a smartphone. When the app is open, it allows the user to take a picture and draw an angle by touching the screen at three points as a reference. Then, the capture (photograph) and the measurement (photograph with angle) are automatically saved in the device (JPEG format, 1.33 MB). The captured image and the measurement made (Fig. 1) enables the user to generate a report with recommendations for clinical use for patients (Fig. 2). The app has been designed and developed by clinical experts with proven experience applying the criteria of evidence-based medicine, so that all clinical recommendations made by the application are supported by medical reference literature. Both measurement and report can be sent by email from the running app.

To obtain information about shoulder disability in pathological subjects, the Spanish version of Disabilities of the Arm, Shoulder and Hand (DASH) [43] and Upper Limb Functional Index (ULFI) [44] questionnaires were filled in by each participant before performing kinematic tasks. The DASH questionnaire is a standardized measure of upper limb symptoms and functional status [45] that consists mainly of a 30-item disability/symptom scale. It has been shown to be valid and reliable in a patient population with various upper limb disorders [46]. ULFI is an upper extremity outcome measure that consists of a 25-item scale, which can be transferred to a 100-point scale. It has also been shown to have strong psychometric properties for reliability and validity [47].

### Procedure

In order to control for potential error introduced by subjects’ positioning and Smartphone placement, the following procedure was used for obtaining images. Participants stood on a mark placed on the floor. Another mark was placed 2 m away in order to maintain the same distance between the subjects and the investigators. Also, shoulder height was measured, so the Smartphone could be positioned at the same height. For this purpose, a rigid measuring tape was used to measure the distance between the ground and the shoulder. This distance was maintained when holding the smartphone and taking the photograph. The Smartphone app mROM corrected anteroposterior tilt on its own, so it was only possible to take the picture when the Smartphone was perpendicular to the ground. Arm abduction was performed in the frontal plane with the elbow extended, the wrist in a neutral position, and the palm of the hand toward the midline at the beginning and end of the movement. Hence, abduction was performed with external rotation because it prevented clearance of the rotator cuff tendons [48]. Participants were told to elevate their arm as much as their shoulder allowed, and to hold their arm still in that maximum arm abduction; they were also told to return to the initial position when the photo was taken. Three photographs taken by the principal examiner were measured for intra-rater reliability. Two of them were taken the first day, and the third one the next day. Another examiner also measured the shoulder on 3 occasions for inter-rater reliability. That is, each examiner took 3 photographs of each subject and determined reference points on 2 separate occasions 1 day apart. Thus, photographic measurements were taken from 3 independent sets of images and, on each measurement occasion, each image was independently examined and an independent measurement produced.

To determinate AAA from the photographs, 3 points were selected by the examiners by touching the screen: humerus-head, middle third of the humerus, and the third one parallel to the center line of the body. mROM used these points to calculate AAA, which is the angle formed by rising the upper limb in a frontal plane. All images were saved in JPEG format (1224 × 1632 pixels). Data was sent to the researchers by email.

For each subject inertial sensors were placed on the half of the body presenting shoulder pathology, located in the middle third of the humerus slightly posterior and in the flat part of the sternum. These surfaces were cleaned with alcohol so each sensor adhered to the skin. To ensure fixation of the sensor to the patient’s skin and prevent slippage, a double-sided adhesive tape was used, as well as an 8 cm wide elastic cohesive (Rapidex ®) to fix to cylindrical body segments (upper arm), and an adhesive bandage 5 cm wide (Strappal ®) in flatter areas of the body (sternum). Inertial values were recorded by kinematic Intersense Server Software, which were subsequently transferred to a Microsoft® Excel 2007 database. In the sensor placed on the humerus, degrees provided by pitch corresponded to humerus abduction. In the sensor placed on the sternum, degrees provided by pitch corresponded to sternum lateral motion. Hence, in order to determinate arm abduction by inertial sensors, degrees recorded in pitch for the sternum were added to those degrees recorded in pitch for the humerus.

All the photographic measurements were taken at the same time as inertial measurement. Therefore direct comparison could be made between inertial measurements and photographs of individual subjects. Both principal examiner and second examiner were physiotherapists with 2 or more years of clinical experience.

At the first day, 2 measurements for one shoulder were recorded using inertial sensors and 2 photographs taken using a Smartphone by two examiners. At the second day, 1 inertial and photographic measurement was taken by each examiner. Repeating both measurements by the principal examiner were used to calculate intra-rater reliability, while measurements by the principal and second examiners were used to calculate inter-rater reliability.

Statistical analysis was performed using data from upper limbs photographs and inertial measurements. Descriptive statistics (mean, standard deviation) using standard procedures were calculated for descriptive and anthropometric variables, as well as for the first measure of AAA by photographs and inertial sensors.

Intra-rater and inter-rater reliability were examined using intraclass correlation coefficients (ICC _2,1_) and *P* value.

For intra-rater reliability, 3 photographic measures taken by the first examiner were compared. For inter-rater reliability, the subject’s best measure from 3 photographs by each examiner was chosen for comparison, as it was understood as the maximum capacity each subject could reach.. Intraclass correlation coefficients (ICC _2,1_) and *P* value were examined. The levels of reliability were excellent (ICC >0.80), good (0.80 > ICC > 0.60), moderate (0.60 > ICC > 0.40), and poor reliability (ICC < 0.40). Very high correlation was represented by *p* value higher than 0.7, whereas coefficients between 0.7 and 0.5 indicated moderate correlation. Values between 0.5 and 0.3 was considered as poor correlation [49]. For criterion validity, *P* values and Pearson correlation coefficients were calculated to examine the association between Smartphone photographic and inertial measurements.

Analysis was performed with SPSS version 22.0 for Windows and Medcalc Software, while data collection used inferential analysis between variables by type and normal. *Kolmogorov*-Smirnov nonparametric tests were used, as determined by the normality of distribution variables. The statistical significance level was set at *P* < 0.05.

## Results

The pathological group consisted of 23 subjects, male/female = 9/14, right-handed/left-handed = 21/2, with unilateral shoulder pain (11 rotator cuff tears, 7 subacromial impingement, 3 supraspinatus tendinopathy, 2 others). 18 right arms and 5 left arms were measured. The healthy group consisted of 14 subjects, male/female = 6/8, all of them right-handed. 12 right arms and 2 left arms were measured.

Descriptive and anthropometric variables are shown in Table 1.

**Table 1:** Descriptive and anthropometric characteristics of sample (*n* = 37)

	Mean ± SD	
	Healthy (*n* = 14)	Pathologic (*n* = 23)
Age (years)	56.14 ± 9.10	52.78 ± 10.02
Weight (Kg)	75.32 ± 14.28	75.24 ± 18.52
Height (m)	1.66 ± 0.09	1.63 ± 0.09
BMI (Kg/m^2^)	27.12 ± 3.85	28.15 ± 6.70
ULFI (0–100)	0	71.82 ± 20.63
DASH (0–100)	0	63.24 ± 18.21

In the overall sample of 37 shoulders, mean ± SD of AAA in the first measure carried out with a Smartphone was 169.07° ± 4.96° for healthy subjects and 93.54° ± 40.88° for pathological subjects. When measuring with inertial sensors mean ± SD of AAA was 154.22° ± 19.27° for healthy subjects and 87.86° ± 47.41 for pathological subjects (Table 2).

**Table 2:** Arm Abduction Angle (degrees) obtained through inertial and photographic measurement in healthy and pathological group

	Minimum		Maximum		Mean ± SD	
	Healthy	Pathologic	Healthy	Pathologic	Healthy	Pathologic
Inertial sensors	128.12	17.53	195.2	186.02	154.22 ± 19.27	87.86 ± 47.41
smartphone	163.87	29.58	179.75	163.37	169.07 ± 4.96	93.54 ± 40.88

For photographic measurements, high values of agreement (Intraclass correlation coefficients (ICC) and 95 % confidence intervals and P) were seen for intra-rater reliability, although these values were higher for the pathological group (Table 3).

**Table 3:** Intra-rater and inter-rater reliability and criterion validity of Smartphone measurement

	Intra-rater reliability ICC _2,1_ (95%CI), *P* value	Inter-rater reliability ICC _2,1_ (95%CI), *P* value	Criterion validity (Pearson r, *P* value)
Healthy group (*n* = 14)	0.780 (0.399, 0.931)	0.492 (0.083, 0.822)	0.400, *p* = 0.198 ns
	*p* = 0.001**	*p* = 0.04*	
Pathologic group (*n* = 23)	0.975 (0.942, 0.989)	0.990 (0.978, 0.996)	0.971, *p* < 0.001**
	*p* < 0.001**	*p* < 0.001**	
Total simple (*n* = 37)	0.988 (0.977, 0.994)	0.994 (0.988, 0.997)	0.964, *p* < 0.001**
	*p* < 0.001**	*p* < 0.001**	

Ns = No significative

* = *p* < 0.05

** = *p* < 0.01

Comparison of measurements taken by 2 different examiners showed very high inter-rater reliability for the pathological group, although it was not reliable for healthy subjects (Table 3).

Validity analysis showed very strong Pearson correlation values for the pathological group and the total sample. However, the Pearson correlation was poor for the healthy group and no significant values were found (Table 3).

## Discussion

To our knowledge, this is the first study to analyze the reliability of a ROM measure using a photography-based Smartphone goniometer in healthy and pathological shoulders. Intra-rater and inter-rater reliability were showed by very high ICC in the total sample. These findings agree precisely with results from other studies investigating photography-based goniometers (ICC greater than 0.93) [50, 51]. However, intra-rater reliability values were better for pathological subjects, and no inter-rater reliability was found for healthy subjects. This fact may be due to a smaller sample in this group. Also, a wider range of motion results in a more heterogeneous distribution, which may affect reliability results. Regarding validity, no relation was found between inertial and image-based measurement. This may be due to the size of the healthy sample (*n* = 14).

Previous studies on shoulder mobility have used photographs to obtain abduction angle. In subjects that underwent shoulder surgery, excellent reliability was found for abduction movement when compared to visual estimation; excellent intra-observer and inter-observer reliability (ICC > 0.9) were also found [50]. The Internet-based goniometer was discovered to be a valid tool for measuring the upper limb range of motion in people who suffered a stroke when compared with a universal goniometer (UG), showing intra- and inter-rater reliabilities (ICC) higher than >0.93 [51]. In our study, an ICC >0.9 was also found for the total sample. However, in the present study, as inertial sensors are an accurate and reliable method for human motion analysis [18] and their use has been supported in upper limbs [19, 20], they were considered an appropriate criterion standard, instead of visual estimation or a UG.In our study, a ICC > 0.9 was also found in the total sample.

As can be appreciated from the bibliography, the concept of obtaining measurements of joint ROM from photographs is not new. In the last decade, it has been investigated for several joints. Hallux valgus measurements using radiography and digital images have been compared, showing also a ICC > 0.9 for inter-rater and intra-rater reliability for photographic measurement, and an acceptable reliability [52]. Hallux valgus angles from foot radiography have been measured by computer-assisted measurements as a criterion standard and compared to an accelerometer-based Smartphone app. For the app, the ICC for inter-observer reliability ranged between 0.56 and 0.93, while the ICC for intra-observer reliability ranged between 0.56 and 0.97, depending on the angle measured or the observer [37]. In the present study, ICC also ranged but showed higher levels, except for inter-rater reliability in the healthy sample, in which the ICC was found to be 0.492. In this case, values ranged depending on the group. Also, knee radiographs were compared to digital photographs for flexion and extension movements, showing near-perfect concordance correlation coefficients, which ranged depending on the method employed or the angle measured [53].

Besides using radiographs, a UG was used as criterion standard, such as for measuring knee angles, and was compared to clinical photographs taken by an Internet-based goniometer, for which the ICC for both reliabilities ranged between 0.96 and 1.00. There were no significant differences found when comparing the UG and the Internet-based goniometer [23]. The UG was also compared to virtual goniometers, which showed higher values of reliability in knee and elbow joints [35]*. A* software programme was shown to be more reliable than the UG when measuring maximal ROM of the knee [54]. Further a Smartphone-based application, showed an ICC for intra and inter-rater reliability higher than 0.956 [55]. Elbow angle measured with a UG was compared with digital photographs. The photography-based method showed an ICC between 0.96 and 0.98 for its validity, and better inter-observer reliability than the UG [35, 36]. A Smartphone image-based app showed an ICC between 0.96 and 0.99 for intra- and inter-rater reliability, respectively, and was shown to be a reliable and useful alternative tool for elbow joint goniometry [29]. Results from the present study are in line with those that showed higher levels of reliability and validity when measuring ROM via Smartphone app. Focusing on ROM provided by image-based app, the range of AAA in our sample of 37 shoulders was 154.44° to 179.75° $\left(\overline{X} = 169.33{}^{\circ}\right)$ for healthy subjects and 29.58° to 169.21° $\left(\overline{X}\kern0.5em =92.69{}^{\circ}\right)$ for pathological shoulders. These results are consistent with previous studies that have investigated AAA: ranging from 20° to 180° for patients who had undergone shoulder surgical procedure [50], mean = 161° of shoulder abduction measured with a goniometer and mean = 162° when measured with an inclinometer in asymptomatic subjects [11], and, in patients with shoulder pathology, the range was from 45° to 180° for visual estimation, from 30° to 170° for goniometry, and from 15° to 160° for still photography [56].

The sample of volunteers recruited were representative of a clinical population, with healthy and pathological shoulders. However, several limitations should be considered in our study. On the one hand, it investigated AAA; however shoulder mobility assessment includes measurements of other movements, such as flexion, extension, scaption, or rotations. Another limitation is that there may be errors when applying these results clinically, including Smartphone placement, subject positioning, and locating landmarks on the screen. However, in order to control for these errors, shoulder height was measured and marks were placed on the floor. Also, one important limitation is its use in obese patients whose surface landmarks may be difficult to accurately pinpoint on the photograph.

As referenced, previous studies have demonstrated that, in some cases, image-based goniometry is more reliable than UG in evaluating ROM. However, traditional manual measure is one of the most widely used methods [2]. More specifically, because of the emergence of new technologies, studies have focused on goniometry via Smartphone app, including image-based measurements, which has shown good reliability and validity, depending on the joint measured and the method employed.

One of the clear benefits of Smartphone photography-based goniometry over other devices is that it represents a portable tool accessible to all. Its non-invasive nature and its cost-effectiveness for investigators and clinicians make it a practical tool to assist with evaluation and treatment compliance.

## Conclusions

This study demonstrates that Smartphone photographs are a reliable and valid method to measure AAA, supporting the use of photography obtained through app for measuring joint ROM. Thus, it offers an interesting alternative to other devices, adding objective information on patients’ shoulder mobility. As clinical implications, this app may assist the medical professional in providing follow-up treatment, allowing to obtain a reliable shoulder measure and to attach by email a clinical report. However, it would be interesting to implement a study with a bigger sample of healthy subjects and to study other arm motions.

## Abbreviations

AAA: arm abduction angle
DASH: disabilities of the arm, shoulder and hand
DOF: degree of freedom
ICC: intraclass correlation coefficients
ROM: range of motion
UG: universal goniometer
ULFI: upper limb functional index

## References

[1] Muir SW, Corea CL, Beaupre L (2010). Evaluating change in clinical status: reliability and measures of agreement for the assessment of glenohumeral range of motion. North Am J Sports Phys Ther NAJSPT.

[2] Boone DC, Azen SP, Lin CM, Spence C, Baron C, Lee L (1978). Reliability of goniometric measurements. Phys Ther.

[3] Fox RF (1917). Physical Remedies for Disabled Soldiers.

[4] Gajdosik RL, Bohannon RW (1987). Clinical measurement of range of motion. Review of goniometry emphasizing reliability and validity. Phys Ther.

[5] Rosén NGNG (1922). A simplified method of measuring amplitude of motion in joints. J Bone Jt Surg.

[6] Mayerson NH, Milano RA (1984). Goniometric measurement reliability in physical medicine. Arch Phys Med Rehabil.

[7] Riddle DL, Rothstein JM, Lamb RL (1987). Goniometric reliability in a clinical setting. Shoulder Measure Phys Ther.

[8] Greene BL, Wolf SL (1989). Upper extremity joint movement: comparison of two measurement devices. Arch Phys Med Rehabil.

[9] MacDermid JC, Chesworth BM, Patterson S, Roth JH (1999). Intratester and intertester reliability of goniometric measurement of passive lateral shoulder rotation. J Hand Ther Off J Am Soc Hand Ther.

[10] Carey MA, Laird DE, Murray KA, Stevenson JR (2010). Reliability, validity, and clinical usability of a digital goniometer. Work Read Mass.

[11] Kolber MJ, Hanney WJ (2012). The reliability and concurrent validity of shoulder mobility measurements using a digital inclinometer and goniometer: A technical report. Int J Sports Phys Ther.

[12] Kolber MJ, Vega F, Widmayer K, Cheng M-SS (2011). The reliability and minimal detectable change of shoulder mobility measurements using a digital inclinometer. Physiother Theory Pract.

[13] Terwee CB, de Winter AF, Scholten RJ, Jans MP, Devillé W, van Schaardenburg D, Bouter LM. Interobserver reproducibility of the visual estimation of range of motion of the shoulder. Arch Phys Med Rehabil. 2005;86:1356–61. 10.1016/j.apmr.2004.12.031 16003664

[14] Mullaney MJ, McHugh MP, Johnson CP, Tyler TF (2010). Reliability of shoulder range of motion comparing a goniometer to a digital level. Physiother Theory Pract.

[15] El-Zayat BF, Efe T, Heidrich A, Wolf U, Timmesfeld N, Heyse TJ, Lakemeier S, Fuchs-Winkelmann S, Schofer MD. Objective assessment of shoulder mobility with a new 3D gyroscope--a validation study. BMC Musculoskelet Disord. 2011;12:168. 10.1186/1471-2474-12-168 PMC3151225 21777447

[16] El-Zayat BF, Efe T, Heidrich A, Anetsmann R, Timmesfeld N, Fuchs-Winkelmann S, Schofer MD. Objective assessment, repeatability, and agreement of shoulder ROM with a 3D gyroscope. BMC Musculoskelet Disord. 2013;14:72. 10.1186/1471-2474-14-72 PMC3614536 23442604

[17] Cuesta-Vargas A, Galán-Mercant A, Williams J. The use of inertial sensors system for human motion analysis. Phys Ther Rev. 2010;15:462–73. 10.1179/1743288X11Y.0000000006 PMC3566464 23565045

[18] Cuesta-Vargas A, Galán-Mercant A, Williams J (2010). The use of inertial sensors system for human motion analysis. Phys Ther Rev.

[19] Kontaxis A, Cutti AG, Johnson GR, Veeger HEJ (2009). A framework for the definition of standardized protocols for measuring upper-extremity kinematics. Clin Biomech Bristol Avon.

[20] Parel I, Cutti AG, Fiumana G, Porcellini G, Verni G, Accardo AP (2012). Ambulatory measurement of the scapulohumeral rhythm: intra- and inter-operator agreement of a protocol based on inertial and magnetic sensors. Gait Posture.

[21] Russell TG (2009). Telerehabilitation: a coming of age. Aust J Physiother.

[22] Theodoros D, Russell T (2008). Telerehabilitation: current perspectives. Stud Health Technol Inform.

[23] Russell TG, Jull GA, Wootton R (2003). Can the Internet be used as a medium to evaluate knee angle?. Man Ther.

[24] Russell TG, Jull GA, Wootton R (2003). The diagnostic reliability of Internet-based observational kinematic gait analysis. J Telemed Telecare.

[25] Russell T (2007). Goniometry via the internet. Aust J Physiother.

[26] Russell TG, Jones AF (2011). Implications of regulatory requirements for smartphones, gaming consoles and other devices. J Geophys Res.

[27] Terry M (2010). Medical Apps for Smartphones. Telemed J E Health.

[28] Steele L, Lade H, McKenzie S, Russell TG (2012). Assessment and diagnosis of musculoskeletal shoulder disorders over the internet. Int J Telemed Appl.

[29] Ferriero G, Sartorio F, Foti C, Primavera D, Brigatti E, Vercelli S (2011). Reliability of a new application for smartphones (DrGoniometer) for elbow angle measurement. PM R.

[30] Jaccard H, Pichonnaz C, Duc C, Lécureux E, Ancey C, Bassin J-P, Aminian K, Farron A, Jolles B, Gleeson N. Validation d’une application smartphone pour l’évaluation de la fonction et de l’amplitude d’élévation de l’épaule. Kinésithérapie Rev. 2014;14:17–8.

[31] Oïhénart L, Duc C, Aminian K (2012). iShould: Functional evaluation of the shoulder using a Smartphone. Gait Posture.

[32] Roldan-Jimenez C, Cuesta-Vargas A, Bennett P (2015). Studying upper-limb kinematics using inertial sensors embedded in mobile phones. JMIR Rehabil Assist Technol.

[33] Shin SH, Ro DH, Lee O-S, Oh JH, Kim SH (2012). Within-day reliability of shoulder range of motion measurement with a smartphone. Man Ther.

[34] Mitchell K, Gutierrez SB, Sutton S, Morton S, Morgenthaler A (2014). Reliability and validity of goniometric iPhone applications for the assessment of active shoulder external rotation. Physiother Theory Pract.

[35] Dunlevy C, Cooney M, Gormley J (2006). Comparing the reliability of virtual goniometry and universal goniometry. Gait Posture.

[36] Blonna D, Zarkadas PC, Fitzsimmons JS, O’Driscoll SW (2012). Validation of a photography-based goniometry method for measuring joint range of motion. J Shoulder Elb Surg Am Shoulder Elb Surg Al.

[37] Walter R, Kosy JD, Cove R (2013). Inter- and intra-observer reliability of a smartphone application for measuring hallux valgus angles. Foot Ankle Surg Off J Eur Soc Foot Ankle Surg.

[38] Cutti AG, Chadwick EK (2014). Shoulder biomechanics and the success of translational research. Med Biol Eng Comput.

[39] Bjelle A (1989). Epidemiology of shoulder problems. Baillieres Clin Rheumatol.

[40] Russell TG (2007). Physical rehabilitation using telemedicine. J Telemed Telecare.

[41] Neer CS (1983). Impingement lesions. Clin Orthop Relat Res..

[42] MacDonald PB, Clark P, Sutherland K (2000). An analysis of the diagnostic accuracy of the Hawkins and Neer subacromial impingement signs. J Shoulder Elb Surg Am Shoulder Elb Surg Al.

[43] Hervás MT, Navarro Collado MJ, Peiró S, Rodrigo Pérez JL, López Matéu P, Martínez Tello I (2006). Spanish version of the DASH questionnaire. Cross-cultural adaptation, reliability, validity and responsiveness. Med Clínica.

[44] Cuesta-Vargas AI, Gabel PC (2013). Cross-cultural adaptation, reliability and validity of the Spanish version of the upper limb functional index. Health Qual Life Outcomes.

[45] Hudak PL, Amadio PC, Bombardier C (1996). Development of an upper extremity outcome measure: the DASH (disabilities of the arm, shoulder and hand) [corrected]. The Upper Extremity Collaborative Group (UECG). Am J Ind Med.

[46] Solway S, Beaton DE, McConnell S, Bombardier C: The DASH Outcome Measure User’s Manual: Disabilities of the Arm, Shoulder and Hand. 2^a^ ed. Inst. for Work & Health; 2002.

[47] Gabel CP, Michener LA, Burkett B, Neller A (2006). The upper limb functional index: development and determination of reliability, validity, and responsiveness. J Hand Ther Off J Am Soc Hand Ther.

[48] Michener LA, McClure PW, Karduna AR (2003). Anatomical and biomechanical mechanisms of subacromial impingement syndrome. Clin Biomech Bristol Avon.

[49] Cohen J: Statistical Power Analysis for the Behavioral Sciences. L. Erlbaum Associates; 1988

[50] O’Neill BJ, O’Briain D, Hirpara KM, Shaughnesy M, Yeatman EA, Kaar TK (2013). Digital photography for assessment of shoulder range of motion: A novel clinical and research tool. Int J Shoulder Surg.

[51] Hoffmann T, Russell T, Cooke H (2007). Remote measurement via the Internet of upper limb range of motion in people who have had a stroke. J Telemed Telecare.

[52] Nix S, Russell T, Vicenzino B, Smith M (2012). Validity and reliability of hallux valgus angle measured on digital photographs. J Orthop Sports Phys Ther.

[53] Naylor JM, Ko V, Adie S, Gaskin C, Walker R, Harris IA, Mittal R. Validity and reliability of using photography for measuring knee range of motion: a methodological study. BMC Musculoskelet Disord. 2011;12:77. 10.1186/1471-2474-12-77 PMC3095577 21496347

[54] Verhaegen F, Ganseman Y, Arnout N, Vandenneucker H, Bellemans J (2010). Are clinical photographs appropriate to determine the maximal range of motion of the knee?. Acta Orthop Belg.

[55] Ferriero G, Vercelli S, Sartorio F, Muñoz Lasa S, Ilieva E, Brigatti E, Ruella C, Foti C. Reliability of a smartphone-based goniometer for knee joint goniometry. Int J Rehabil Res Int Z Für Rehabil Rev Int Rech Réadapt. 2013;36:146–51. 10.1097/MRR.0b013e32835b8269 23196790

[56] Hayes K, Walton JR, Szomor ZR, Murrell GA (2001). Reliability of five methods for assessing shoulder range of motion. Aust J Physiother.

